# Inhibition of L-Cysteine on the Browning of Fresh Wet Noodles

**DOI:** 10.3390/foods10061156

**Published:** 2021-05-21

**Authors:** Xin-Ting Wu, Xiao-Na Guo, Ke-Xue Zhu

**Affiliations:** State Key Laboratory of Food Science and Technology, School of Food Science and Technology, Jiangnan University, 1800 Lihu Avenue, Wuxi 214122, China; 18762691378@163.com (X.-T.W.); kxzhu@jiangnan.edu.cn (K.-X.Z.)

**Keywords:** L-cysteine, browning, mechanism, inhibition, FWN

## Abstract

This research explored the effect of L-cysteine on the browning of fresh wet noodles (FWN). With the increasing addition of L-cysteine (0.02–0.1%), the ΔL* decreased and Δa*, Δb* increased. The L-cysteine could reduce the pH value and polyphenol oxidase (PPO) activity and increase the retention rate of polyphenol of FWN. It suggested that L-cysteine could inhibit the browning of FWN by decreasing pH value, PPO activity, and the oxidation of polyphenols. In the in vitro PPO solution, the inhibitory effect of L-cysteine on PPO activity was related to the decrease in pH and the ability of chelating Cu^2+^. According to UPLC-TOF-MS analysis, L-cysteine could reduce the generation of browning products, which suggested that L-cysteine could react with the browning intermediate product (quinone) and generate a light-colored substance (-C_9_H_10_NO_4_S). L-cysteine effectively inhibited the browning of FWN and had the potential to be used in noodle industry.

## 1. Introduction

Noodles are one of the staple foods in Asian counties, composed of wheat flour, water and additives (salt and sodium carbonate). Noodles are mainly classified into dried noodles, semi-dry noodles and fresh wet noodles (FWN) according to the difference in water content [1]. FWN is getting increasingly popular among consumers due to their unique taste compared to dried noodles [2]. However, due to the high moisture content, the overall color of FWN changes from bright milky white or light yellow to gray or brown during storage, affecting its quality and sale [3].

The browning of FWN is related to many factors [4]. Previous studies have confirmed that polyphenol oxidase (PPO) played an important role in the browning of alkaline wet noodles [5]. PPO and phenols in wheat kernels were mainly concentrated in the bran layer [6,7,8]. After the milling process, the tissues of the bran are inevitably mixed into the flour. PPO can interact with polyphenols, producing brown substances, causing browning of noodles [9]. During the processing and storage of flour or FWN, wheat PPO maintains high activity, and it is difficult to inactivate PPO by heat treatment without destroying gluten of flour [6,7]. Asenstorfer [10,11] proposed that the browning degree of the yellow alkaline noodles within 24 h was positively correlated with protein content by kinetic studies. There are many external factors that affect browning of FWN, including the amount of water added, pH, vacuum, storage temperature, and browning inhibitors. The vacuum mixing could reduce the browning of FWN and increase the brightness of the initial noodles [12].

Many studies have focused on the inhibition of browning of noodles. When the whole wheat flour was treated with 700 W microwave power for 90 s, the PPO activity of whole wheat flour was almost inhibited, and the browning degree of the fresh whole wheat noodles was significantly reduced [13]. However, microwave processing of whole wheat flour changed the flour characteristics and affected the quality of noodles. Brutsch proposed that the browning of pastry dough could be inhibited by combination of white wine and fresh lemon juice. However, it had a complex flavor, had high cost, and was not suitable for the commercial production of FWN [14]. The combination of glucose oxidase, papain, and xylanase could reduce the browning of noodles and improve the rheological characteristics of dough [15].

L-cysteine is a safe and natural amino acid. As a food additive, it is widely used in food processing. L-cysteine could be an effective anti-browning agent of Amasya apple juice due to the ability of inhibiting PPO activity [16]. Naim reported that the addition of L-cysteine could reduce the degradation of ascorbic acid in orange juice and inhibit browning [17]. After being treated with a mixed solution of L-cysteine, complex phosphate and fatty acid sucrose ester, the browning degree of cooked rice during storage was reduced [18]. The antioxidant capacity of fresh-cut lettuce dipped with L-cysteine increased, and the degree of browning decreased significantly [19]. However, the causes and mechanism of L-cysteine inhibiting food browning have not been deeply studied. L-cysteine was a non-competitive inhibitor of PPO from Whangkeumbae pear [20], while some studies showed that in the enzymatic browning reaction mixture, L-cysteine could transform the browning products to colorless substances, reducing the degree of enzymatic browning [21].

L-cysteine was often used in dough to improve its rheological characteristics [22]. Yadav proposed that the discoloration of dough could be prevented by L-cysteine (90 ppm), which showed that L-cysteine could be used as a potential browning inhibitor for pasta products [23]. However, the inhibitory effect and mechanism of L-cysteine on the browning of FWN have not been studied. Therefore, the objectives of this work were to: investigate the impact of L-cysteine addition on the browning of FWN; determine the pH, PPO activity, and retention rate of polyphenol of FWN; explore the relationship between the changes in PPO activity caused by L-cysteine and pH value or Cu^2+^ chelation in PPO solution; and analyze PPO browning products of in vitro PPO-catechol system by the UPLC-TOF-MS.

## 2. Materials and Methods

### 2.1. Materials

L-cysteine was purchased from Sinopharm Chemical Reagent Co., Ltd. (Shanghai, China). Copper acetate (PubChem CID: 24622, AR, 99%) and catechol (PubChem CID: 24848193, AR, 99%) were purchased from Sigma-Aldrich Inc. (New York, NY, USA). Wheat flour (protein content of 12.36%, ash content of 0.59%, and water content of 12.17%) was purchased from COFCO Group Ltd. (Beijing, China). Formic acid and acetonitrile used in the mobile phase were of HPLC grade. The other chemicals used were of analytical grade.

### 2.2. Preparation of FWN

FWN were prepared according to the method of Li [12]. Briefly, 200 g of flour, 2 g of sodium chloride, and 66 mL of distilled water with or without L-cysteine were mixed into a needle dough mixer (Kitchen Aid, St. Joseph, MI, USA) for 7 min. The dough was stored at 25 °C for 30 min, and then, the dough was rolled to form dough sheets (thickness of 1.0 mm) by a noodle maker, a laboratory sheeting machine (JMTD 168/140, Dongfu Jiuheng Instrument Technology Co., Ltd., Beijing, China). The noodle sheets were then cut into round pieces with a radius of 5 cm. Finally, the round pieces were put into bags to maintain the moisture content at a constant value, and then, the samples were stored at 25 °C for 24 h.

### 2.3. Determination of the Color of FWN

The color of FWN was measured by the method of Asenstorfer [10]. After being stored for 24 h, the values of L*, a*, and b* of FWN were determined by a chromameter (CR-400, Konica Minolta Holdings Inc., Tokyo, Japan). L*, a*, and b* represented brightness (0–100), red-green coordinates, and blue-yellow coordinates of samples, respectively. Samples with a higher value of L* were brighter, and samples with a higher value of a* were redder. Moreover, samples with a higher value of b* were more yellow. Each noodle sheet was measured 6 times. The changes of color (ΔL*, Δa*, and Δb*) were calculated by subtracting the readings on 24 h from the readings on 0 h.

### 2.4. Analysis of Polyphenol Oxidase Activity of FWN

PPO activity was measured based on the method of Yoruk [24]. FWN was freeze-dried (−45 °C, 48 h), ground, and sieved (100 mesh). The sample was used for the determination of PPO activity. Briefly, 10 mL of phosphate buffer (0.1 M, pH 6.0) was mixed with 1 g of sample in a tube, which was shaken continuously for 24 h (4 °C). After that, the mixed solution was centrifuged at 10,000 rpm for 20 min (4 °C). The supernatant was collected and used for the analysis of PPO activity. Further, 1.25 mL of supernatant and 0.25 mL of catechol (0.1 M) were mixed in the 24-well microtiter plate. Then, the 24-well microtiter plate was put into Enzyme standard instrument (M5, Molecular Devices Inc., San Jose, CA, USA). The absorbance of the mixture was recorded every 20 s at 420 nm for 3 min. The change in 0.001 in absorbance per gram of sample within 1 min was defined as 1 unit (U/g^−1^·min^−1^).

### 2.5. Determination of Content of Polyphenols Retention Rate in FWN

The polyphenols content of FWN was determined by the method of Yu [25]. FWN was freeze-dried (−45 °C, 48 h), ground, and sieved (100 mesh). The extractions of polyphenols were obtained by mixing 0.5 g of sample and 5 mL of 70% pre-heated methanol (70 °C) into the brown centrifuge tube (10 mL) and shaking for 10 min. Next, a brown centrifuge tube was centrifuged (4000 rpm, 10 min, and 4 °C). Then, the above operations were repeated twice and two supernatants were collected into tubes and diluted to 10 mL. Precisely, 1 mL of the above diluted extract and 4 mL of Folin–Ciocalteu reagent (10% *v*/*v*) were mixed into a 10 mL brown tube for 3 min. Then, 5 mL of 7% sodium carbonate solution was added to the above brown tubes. After 1 h, the absorbance of the mixture at 765 nm was determined. The reagent blank was 70% of methanol instead of supernatants solution. For the standard curve, 1 mL of supernatant was replaced with 1 mL of gallic acid solution of different concentrations, and the above determination steps for polyphenols were repeated. The standard curve was obtained based on the concentration gallic acid solutions and corresponding values absorbance. The retention rate of polyphenol was presented by the ratio of the polyphenols content of the FWN after 24 h to the polyphenols content of the FWN stored for 0 h.

### 2.6. Preparation of PPO Solution

The PPO solution was extracted from wheat bran (COFCO Group Ltd. Beijing, China). Wheat bran was prepared by grinding and sieving (100 mesh). Then, wheat bran and deionized water were mixed at a ratio of 1:10, and the mixture was continued to shake for 4 h. After that, the above mixture was centrifuged at speed of 4000 rpm for 20 min. The supernatant (crude enzyme solution) was collected and used as PPO solution.

### 2.7. Analysis of the Inhibitory Effect of L-Cysteine on the PPO Activity by Reducing pH

The relationship between changes in PPO activity and pH values caused by L-cysteine was analyzed according to the method of Tsouvaltzis [26]. PPO solutions obtained by the method in Section 2.6 were used as material. L-cysteine (0, 0.04%, and 0.1%) was added to the PPO solution, and the pH and PPO activity of solutions were measured after mixing. In order to explore whether L-cysteine inhibits PPO activity by lowering pH, the following experiment was designed. L-cysteine (0, 0.04%, and 0.1%) was added to the PPO solution and completely dissolved. Then, the pH values of the PPO solutions with L-cysteine were adjusted to the pH of PPO solutions without L-cysteine by 0.1 mol/L NaOH. After that, the PPO activity of PPO solutions was measured again.

### 2.8. Analysis of the Inhibitory Effect of L-Cysteine on the PPO Activity by Chelating Cu^2+^

The experimental treatments were set up and analyzed based on the report of Yourk [24]. For this, 0%, 0.04%, and 0.1% of L-cysteine were mixed with 10 mL PPO solutions separately. Among them, PPO solution with 0% L-cysteine was control, and PPO solutions with 0.04% and 0.1% of L-cysteine were treatment groups. First, control and treatment groups were prepared, and the PPO activities of all groups were determined. In order to explore whether L-cysteine inhibits polyphenol oxidase by chelating Cu^2+^, the following experiment was designed. Furthermore, 0%, 0.04%, and 0.1% of L-cysteine were mixed with 10 mL PPO solutions separately and completely dissolved. Then, 0%, 0.5%, and 1% of copper acetate was added to control and treatment groups. After that, the PPO activities of all groups were analyzed. The method of determination of PPO activity is reported in Section 2.4.

### 2.9. Preparation of PPO-Catechol System

The PPO-catechol system was prepared to simulate the PPO browning reaction in FWN. The PPO-catechol system was prepared according to Zhao [27]. For this, 10 mL of the PPO solution, 10 mL of water (with or without L-cysteine), and 2 mM catechol (substrate) were mixed and the PPO-catechol system was established. The PPO-catechol system was incubated at 37 °C for 20 min to accelerate the PPO catalytic reaction, and then, it was stored at 25 °C.

### 2.10. Analysis of the Browning Products by UPLC-TOF-MS

The PPO-catechol systems with or without L-cysteine were stored at 25 °C for 48 h. Then, 5 mL of hydrochloric acid (HCl) was added to terminate the reaction.

After 48 h reaction, the PPO-catechol systems were clarified by passing through membrane filters with the aperture of 0.22 µm. Through ultra-performance liquid chromatography-time off flight mass spectrometry (UPLC-TOF-MS, Waters Inc., Milford Massachusetts, MA, USA), which was equipped with ACQUITY UPLC BEH C18 2.1 × 150 mm 1.7 µm, the components of PPO-catechol systems were determined. The conditions of analyzing browning products by UPLC were based on the measure of Bornik and Kroh [28]. The elution procedure of the mobile phase was 80% acetonitrile, 20% formic acid, and 0.1% ammonia at the flow rate of 0.3 mL/min. The temperature of the column was 45 °C and the injection volume was 5 μL.

TOF-MS of browning products was conducted according to the method of Zhao [27]. TOF-MS was performed with Waters MALDI SYNAPT Q-TOF MS (Waters Inc., Milford Massachusetts, MA, USA) at ESI mode, which was under the conditions of a capillary of 3.5 kV, a cone of 30 V, a source block temperature of 100 °C, a desolvation temperature of 400 °C, a desolvation gas flow of 700 L/h, a cone gas flow of 50 L/h, and a collision energy of 6/20 eV. The range of molecular weight was 20–2000 and the detector was 1800 v. The spectra and elemental composition analysis were performed using the software MassLynx V4.1 (Waters Inc., Milford Massachusetts, MA, USA).

### 2.11. Statistical Analysis

The data were reported as mean ± standard deviation (SD). Each experiment was repeated at least three times. Data obtained were analyzed using the software SPSS 22.0 (SPSS Inc., Chicago, IL, USA) for Windows. Analysis of variance (ANOVA) was conducted at a 95% confidence level.

## 3. Results

### 3.1. The Inhibitory Effect of L-Cysteine on the Browning of FWN

**Figure 1:**
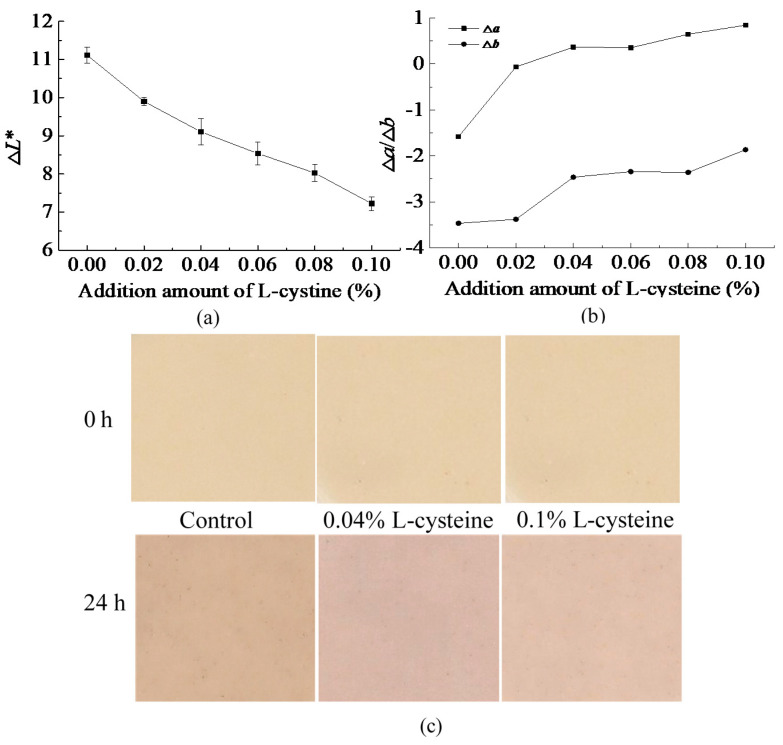
Effect of L-cysteine on the browning of FWN: (**a**) ΔL* of FWN, (**b**) Δa*/Δb* of FWN, and (**c**) pictures.

The inhibitory effect of L-cysteine on the browning of FWN was shown in Figure 1. The L* represented brightness of FWN. The ∆L* represented the difference between the initial L* value of the FWN and the L*value of noodles stored for 24 h (L*_0_–L*_24h_). The ∆L* of FWN gradually decreased with the increase in the addition amount of L-cysteine (Figure 1a). Moreover, the ∆L* of FWN with L-cysteine was smaller than that of control. However, the ∆L* also reflected the browning degree of FWN, and the larger the ∆L* value, the more serious the browning of FWN was found. The results in Figure 1a indicate that the browning degree of the FWN with the L-cysteine treatment was lower than that of control. It also could be seen from Figure 1c that after 24 h of storage, the color of the FWN became darker and the brightness decreased, causing ∆L* > 0. According to Figure 1c, after 24 h storage, the FWN with L-cysteine treatment was brighter than control (0% L-cysteine), especially in 0.1% L-cysteine addition. It indicated that the addition of L-cysteine could reduce the browning degree of FWN. Researchers reported that the addition of L-cysteine also could decrease the browning degree of whole wheat flour dough sheet [23].

The values of a* and b* represented greenness-red and blue-yellowness, respectively. ∆a*, ∆b* represented the difference between the initial a*, b* values of the FWN and the a*, b* values of storage for 24 h. If ∆a*, ∆b* < 0, it meant that the color of the sample shifted toward red and yellow, respectively, otherwise, it shifts toward green and blue. As shown in Figure 1b, after 24 h storage, ∆b* < 0. It meant that the color of the FWN shifted toward yellow. With the addition of L-cysteine, ∆b* gradually became larger and was close to zero during 24 h storage, indicating that the color of FWN shifted less toward yellow and the change of yellowness in FWN was reduced. For control, ∆a* > 0, the color of the sample shifted toward red after 24 h. As the addition amount of L-cysteine increased, the ∆a* also increased. It meant that the color of FWN shifted less toward red, tending to shift toward green. PPO played an important role in food browning. Bayindirli reported that L-cysteine could be an effective anti-browning agent of Amasya apple juice due to the ability of inhibition of PPO activity [16]. It was speculated that the effect of cysteine on the browning of FWN might be related to PPO activity.

### 3.2. Effect of L-Cysteine on the pH, PPO Activity, and Retention Rate of Polyphenols of FWN

Effects of L-cysteine on the pH and PPO activity of FWN are shown in Table 1. With the addition amount of L-cysteine increase, the pH of FWN significantly (*p* < 0.05) decreased (Table 1). When the addition amount of L-cysteine was 0.1%, the pH of the FWN decreased from 6.03 (control) to 5.67. According to the literature, for L-cysteine, the PKa (PKa = −logKa) of -COOH and -NH_2_ were 1.96 (PKa_1_) and 10.28 (PKa_2_), respectively [29]. Ka represents dissociation constant and indicates the dissociation ability. The dissociation ability of -COOH (Ka_1_: 0.011) is greater than that of -NH_2_ (Ka_2_: 5.25 × 10^−11^), indicating that the amount of -COO^−^ produced by dissociation of -COOH is greater than the amount of -NH_3_^+^ produced by combination of -NH_2_ and H^+^, so L-cysteine solution is slightly acidic, which leads to the decrease in pH of noodles. Preview research showed that the pH of the noodles significantly affected the browning of FWN, and the browning degree of FWN was reduced at low pH values [6,12]. It was speculated that the inhibitory effect of L-cysteine on FWN might be related to the decrease in pH value.

As the addition amount of L-cysteine increased, the PPO activity of the FWN gradually decreased (Table 1). It could be seen that L-cysteine reduced the browning degree of FWN by inhibiting the PPO activity. PPO could catalyze the oxidation of polyphenols to produce dark substances, causing food browning [6]. The degree of overall browning in the FWN during 24 h storage was positively correlated with PPO activity by kinetic studies [5]. Alandes reported that L-cysteine could reduce the PPO activity and inhibit browning in fresh apples [30]. It was concluded that the inhibitory effect of L-cysteine on the browning of FWN was achieved by reducing the pH and PPO activity.

The effect of L-cysteine on the retention rate of polyphenols on FWN is shown in Table 1. The retention rates of polyphenol of FWN in the control group and the L-cysteine treatment group were less than 100% after storage for 24 h. It indicated that after 24 h storage, the polyphenols were oxidized to produce brown substances, and the content of polyphenol was reduced. With the addition amount of L-cysteine increasing, the retention rate of polyphenol of FWN was significantly increased. The retention rate of polyphenol of FWN with L-cysteine was higher than that of control (*p* < 0.05). When the addition amount of L-cysteine was 0.1%, the retention rate of polyphenol of the FWN changed from 78.44% (control) to 82.2%. It could be concluded that L-cysteine reduced the oxidation of polyphenols and increased the retention rate of polyphenol.

The addition of L-cysteine increased the retention rate of polyphenol of FWN, which indicated that the oxidation of polyphenols could be inhibited by L-cysteine. In the process of PPO browning, the phenolic compounds were oxidized to benzoquinones under the catalysis of PPO, and then benzoquinones were immediately polymerized with phenolic compounds and proteins to form dark substances [14,31]. Caffeic acid, chlorogenic acid, catechins, tyrosine, and other phenolic substances were concentrated in wheat bran, and they could be substrates for the enzymatic browning [6]. The browning of potato was significantly related to the change in polyphenol content during storage. Tyrosine and chlorogenic acid were the main substrates and caused enzymatic browning of potato [32]. It could be concluded from the above results that L-cysteine could reduce the oxidation of polyphenols and increase retention rate of polyphenols in FWN during storage, thereby inhibiting browning of FWN.

### 3.3. Relationship between the Changes in PPO Activity Caused by L-Cysteine and pH Value

The addition of L-cysteine could decrease the PPO activity and pH value of FWN. Therefore, the PPO solution was used to explore the relationship between the changes in PPO activity caused by L-cysteine and the pH value. The addition of L-cysteine reduced the pH of PPO solutions, and the pH values were significantly decreased as the concentration increased (Table 2 Group 1). When the addition amount of L-cysteine was 0, 0.04%, and 0.1%, the pH values of the PPO solutions were 6.35, 5.42, 4.73, respectively. The addition of L-cysteine obviously decreased the PPO activity of PPO solutions. PPO activity was significantly related to pH value [31]. Current research suggests that some organic acids (malic acid and citric acid) could effectively inhibit PPO activity by lowering pH [32,33].

In order to explore whether the inhibitory effect of L-cysteine on PPO activity was related to the decrease in pH, the pH of PPO solutions treated with L-cysteine was adjusted to the pH 6.35 (equal to pH of control) using 0.1 mol/L sodium hydroxide (NaOH) solution. When pH value of the solution with 0.04% L-cysteine was adjusted from pH 5.42 to 6.35, the PPO activities changed from 243.47 to 275.69 U·g^−1^·min^−1^ (Table 2 Group 2). While pH value of the solution with 0.1% L-cysteine was adjusted from pH 4.73 to 6.35, the PPO activity changed from the 198.97 to 221.58 U·g^−1^·min^−1^ (Table 2 Group 2). After being adjusted to pH 6.35, PPO activities of the solutions with L-cysteine recovered to some extent, but did not reach the level of the control. Especially for the solution with higher L-cysteine, it was not easy to recover the PPO activity after being adjusted to pH 6.35.

L-cysteine could decrease the pH value and inhibit PPO activity of the solutions.

In order to explore whether the L-cysteine inhibited PPO activity only by decreasing pH value, the pH value of the solutions was adjusted by HCl to pH 5.42 and 4.73 separately (equal to pH of group 1) and the PPO activities of the solutions were determined. PPO activities of the solutions were 280 and 230.71 U·g^−1^·min^−1^ (Group 3), which was lower than that of control and higher than that of solutions with L-cysteine (Group 1). When the solutions were adjusted to 6.35 by NaOH, the activities increased to 340.83 and 340.11 U·g^−1^·min^−^^1^ significantly (Group 4) and reached the level of the control, which showed that PPO activities of the solutions with HCl were largely recovered by adjusting the pH. L-cysteine treatment had a better inhibitory effect on wheat PPO than HCl treatment at the same pH value, and the PPO activities of solutions with L-cysteine were partly recovered by adjusting the pH. The results suggested that the decrease in PPO activity by L-cysteine treatment was not totally affected by pH value.

### 3.4. Relationship between the Changes in PPO Activity Caused by L-Cysteine and Chelating Cu^2+^

**Figure 2:**
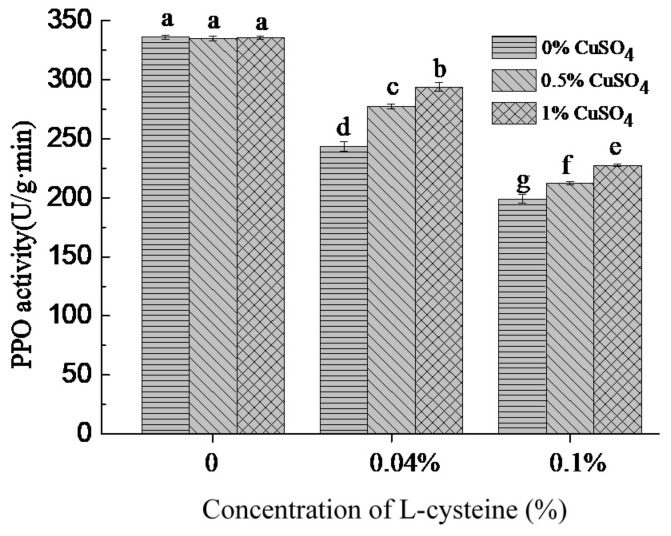
Effect of copper acetate on PPO activity of PPO solutions mixed with L-cysteine. Different lowercase letters show significant differences (*p* < 0.05).

PPO was a kind of metal enzyme. Copper ion exists in its active center and was closely related to PPO activity [34]. To determine whether L-cysteine inhibited PPO activity in PPO solutions through Cu^2+^ chelation, the PPO activities of control (without cysteine) group and treatment group (with L-cysteine) were determined. As was shown in Figure 2, in the control group, the PPO activities of PPO solutions treated with 0.5% and 1.0% copper acetate were not significantly changed compared with the 0% copper acetate, whereas in the treatment groups, higher PPO activities were observed with the increase in concentration of copper acetate, but the PPO activities did not reach to the level of control. These results indicated that the PPO activity of PPO solutions pre-treated with L-cysteine could be restored to some extent by adding copper acetate. The inhibition of PPO activity by L-cysteine could be related to its ability of chelating Cu^2+^.

Some studies pointed out that the -SH group of L-cysteine had a good binding ability to copper ions and could react with copper ions of the active center of polyphenol oxidase and reduce the PPO activity [35]. According to previous research, organic acids and organic acid salts were good metal chelating agents and used to inhibit browning of food [26,32]. The inhibition of PPO by oxalic acid was due to its binding with copper to form an inactive complex, and the inhibition was characterized as noncompetitive [24].

### 3.5. The Browning Products Analysis by UPLC-TOF-MS in PPO-Catechol System

**Figure 3:**
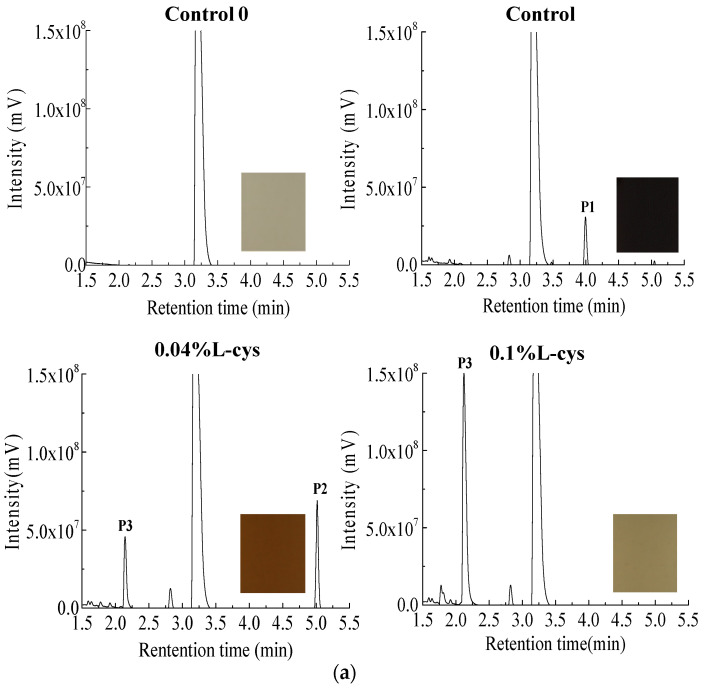

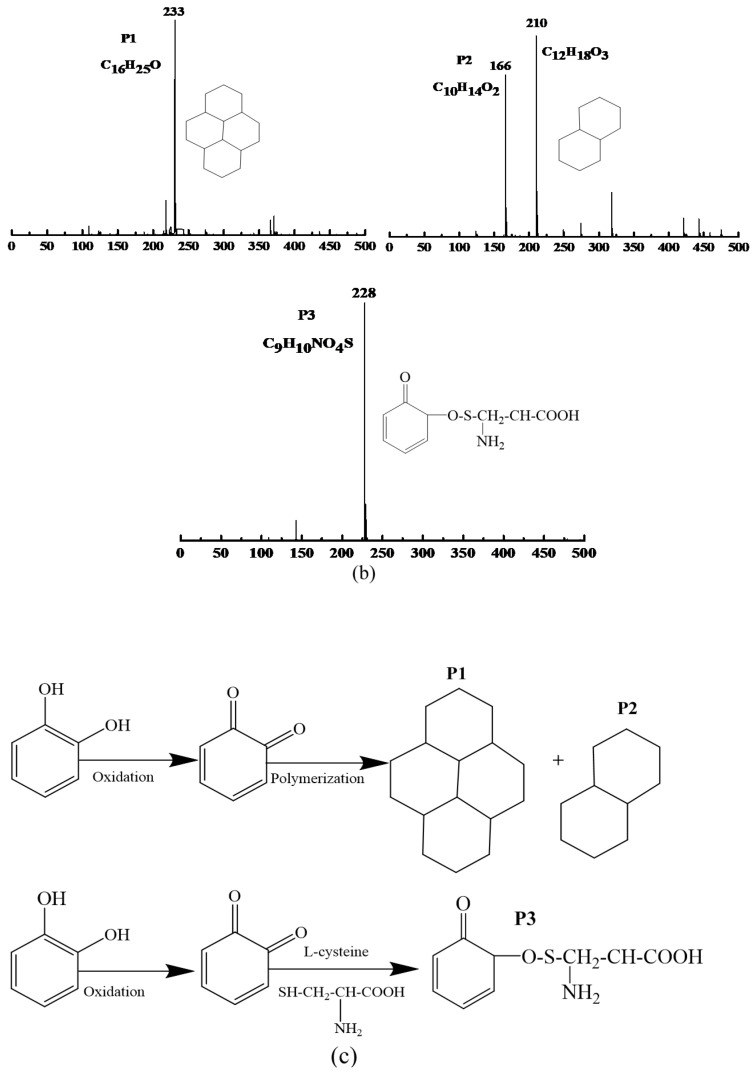
Analysis of enzymatic browning products in PPO-catechol system: (**a**) UPLC spectra in PPO-catechol system, (**b**) mass spectrometry analysis, and (**c**) inhibitory mechanism of L-cysteine on enzymatic browning.

In order to explore the effect of L-cysteine on PPO browning products of FWN, the PPO-catechol system was used to simulate the PPO browning of FWN. The enzymatic browning products were analyzed by HPLC-TOF-MS (Figure 3). After 48 h reaction, the color of the PPO-catechol system in control group became darker, which suggested that colored substances were produced in the system. Compared with the control group, the color of the system with 0.04% and 0.1% L-cysteine was lighter, especially in 0.1% L-cysteine. It indicated that L-cysteine could significantly affect the generation of PPO browning products.

After 48 h reaction, one main peak was produced in control group at a retention time of 3.99, which was marked as P1. From the change of color and peak area of the PPO-catechol systems, it could be inferred that P1 might be a dark-colored product of PPO browning. Compared with the control group, there were two main peaks produced (P2 with retention time of 5.05 and P3 with retention time of 2.28) in the 0.04% treatment group and one main peak P3 produced in the 0.1% treatment group (Figure 3a). It was speculated that P1 might be the product of PPO browning, while the appearance of P2 and P3 might be related to the addition of L-cysteine. Therefore, the molecular structure of P1, P2, and P3 has been analyzed.

The molecular structure of P1, P2, and P3 was analyzed by mass spectrums and software MassLynx V4.1 (Waters Inc., Milford, MA, USA) (Figure 3b). The P1 was -C_16_H_25_O (*m*/*z* 233), and it might be a tetrameric compound, which was generated by the oxidative polymerization of catechol. The P2 possessed -C_10_H_14_O_2_ (166), a dimeric compound that produced by oxidative polymerization of catechol, or -C_12_H_18_O_3_ (210), which might be dimerized catechol residue. The P3 was identified as -C_9_H_10_NO_4_S (*m*/*z* 288) that might be six-carbon compounds derivatives, which was related to the reaction of L-cysteine and phthaloquinone. PPO catalyzed the dehydrogenation of catechol to 1,2-benzoquinone, which was extremely active. Once formed, it was immediately auto-oxidized and polymerized, causing brown substances [36,37].

The specific inhibitory pathway by which L-cysteine inhibited enzymatic browning was speculated and shown in Figure 3c. The catechol was catalyzed by polyphenol oxidase to o-phthaloquinone. Then, o-phthaloquinone was further oxidized and polymerized, and a brown compound (P1) was formed. However, L-cysteine could react with the intermediate product (phthaloquinone) of the PPO browning reaction, thus preventing further oxidation and polymerization of quinone and producing stable light-colored substance (P3). In the model system of PPO-chlorogenic acid, the results indicated that the mechanism of L-cysteine on browning inhibition was related to the formation of a thiol-conjugated reaction product [38].

## 4. Conclusions

L-cysteine could significantly inhibit the browning of FWN. L-cysteine could reduce the browning degree of FWN by reducing pH value, inhibiting the activity of PPO, and oxidation of polyphenols. In the in vitro PPO-catechol system, the underlying mechanism of L-cysteine in inhibiting the PPO was discussed. Results showed that L-cysteine effectively inhibited PPO activity by lowering pH and chelating copper ions. Moreover, L-cysteine could react with intermediate products of enzymatic browning (quinone), transforming intermediate products into some light-colored products (-C_9_H_10_NO_4_S). L-cysteine could inhibit the browning of FWN, suggesting it might be a promising anti-browning agent for the food industry.

## Figures and Tables

**Table 1:** pH value, PPO activities, and retention rate of polyphenols of FWN.

Addition Amount of L-Cysteine (%)	pH Value	PPO Activity (U/g·min)	Retention Rate of Polyphenols (%)
0	6.02 ± 0.02 ^a^	318.32 ± 2.99 ^a^	78.44 ± 0.17 ^f^
0.02	5.97 ± 0.02 ^b^	288.05 ± 7.42 ^b^	79.41 ± 0.08 ^e^
0.04	5.88 ± 0.01 ^c^	270.78 ± 7.15 ^c^	80.18 ± 0.13 ^d^
0.06	5.76 ± 0.01 ^d^	268.18 ± 5.09 ^c^	80.79 ± 0.09 ^c^
0.08	5.62 ± 0.02 ^e^	250.69 ± 8.90 ^d^	81.24 ± 0.11 ^b^
0.10	5.57 ± 0.02 ^f^	240.09 ± 10.97 ^d^	82.20 ± 0.11 ^a^

^a–f^ Different lowercase letters mean a significant (*p* < 0.05) difference in values of the same column.

**Table 2:** pH value and PPO activities of PPO solutions.

Group	Preparation of PPO Solutions	Final pH	PPO Activity (U·g^−1^·min^−1^)
Control		6.35	336.02 ± 1.66 ^a^
Group 1	0.04% L-cysteine added	5.42	243.47 ± 3.96 ^c^
	0.1% L-cysteine added	4.73	198.97 ± 3.92 ^f^
Group 2	0.04% L-cysteine added, mixed, and pH adjusted with NaOH	6.35	275.69 ± 1.78 ^b^
	0.1% L-cysteine added, mixed, and pH adjusted with NaOH	6.35	221.58 ± 1.86 ^e^
Group 3	HCl added	5.42	280.14 ± 3.62 ^b^
	HCl added	4.73	230.71 ± 2.63 ^d^
Group 4	HCl added, mixed, and pH adjusted with NaOH	6.35	340.83 ± 3.14 ^a^
	HCl added, mixed, and pH adjusted with NaOH	6.35	340.11 ± 3.86 ^a^

^a–f^ Different lowercase letters mean a significant (*p* < 0.05) difference in values of the same column.

## Data Availability

The data that support the findings of this study are available on request from the corresponding author. The data are not publicly available due to privacy or ethical restrictions.
